# Huzhangoside A Suppresses Tumor Growth through Inhibition of Pyruvate Dehydrogenase Kinase Activity

**DOI:** 10.3390/cancers11050712

**Published:** 2019-05-23

**Authors:** Choong-Hwan Kwak, Jung-Hee Lee, Eun-Yeong Kim, Chang Woo Han, Keuk-Jun Kim, Hanna Lee, MyoungLae Cho, Se Bok Jang, Cheorl-Ho Kim, Tae-Wook Chung, Ki-Tae Ha

**Affiliations:** 1 Korean Medical Research Center for Healthy Aging, School of Korean Medicine, Pusan National University, Yangsan, Gyeongsangnam-do 50612, Korea; hahaaaa@nate.com (C.-H.K.); desire1213@naver.com (J.-H.L.); eylove0822@hanmail.net (E.-Y.K.); 2 Department of Korean Medical Science, School of Korean Medicine, Pusan National University, Yangsan, Gyeongsangnam-do 50612, Korea; 3 Department of Molecular Biology, College of Natural Sciences, Pusan National University, Geumjeong-gu, Busan 46241, Korea; hotorses@naver.com (C.W.H.); sbjang@pusan.ac.kr (S.B.J.); 4 Department of Clinical Pathology, TaeKyeung University, Gyeongsan 38547, Korea; biomed@tk.ac.kr; 5 National Development Institute of Korean Medicine, Gyeongsan, Gyeongsanabuk-do 38540, Korea; iiihanna@nikom.or.kr (H.L.); meanglae@nikom.or.kr (M.C.); 6 Department of Biological Science, Sungkyunkwan University, Suwon, Kyunggi-do 16419, Korea; chkimbio@skku.edu

**Keywords:** Huzhangoside A, Warburg effect, Pyruvate dehydrogenase kinase, Glycolysis, Apoptosis

## Abstract

Aerobic glycolysis is one of the important metabolic characteristics of many malignant tumors. Pyruvate dehydrogenase kinase (PDHK) plays a key role in aerobic glycolysis by phosphorylating the E1α subunit of pyruvate dehydrogenase (PDH). Hence, PDHK has been recognized as a molecular target for cancer treatment. Here, we report that huzhangoside A (Hu.A), a triterpenoid glycoside compound isolated from several plants of the *Anemone* genus, acts as a novel PDHK inhibitor. Hu.A was found to decrease the cell viability of human breast cancer MDA-MB-231, hepatocellular carcinoma Hep3B, colon cancer HT-29, DLD-1, and murine lewis lung carcinoma LLC cell lines. The activity of PDHK1 was decreased by Hu.A in both in vitro assays and in vivo assays in DLD-1 cells. Hu.A significantly increased the oxygen consumption and decreased the secretory lactate levels in DLD-1 cells. In addition, Hu.A interacted with the ATP-binding pocket of PDHK1 without affecting the interaction of PDHK1 and pyruvate dehydrogenase complex (PDC) subunits. Furthermore, Hu.A significantly induced mitochondrial reactive oxygen species (ROS) and depolarized the mitochondrial membrane potential in DLD-1 cells. Consistently, when Hu.A was intraperitoneally injected into LLC allograft mice, the tumor growth was significantly decreased. In conclusion, Hu.A suppressed the growth of tumors in both in vitro and in vivo models via inhibition of PDHK activity.

## 1. Introduction

Pyruvate dehydrogenase kinase (PDHK) is a mitochondrial enzyme that suppresses the activity of pyruvate dehydrogenase complex (PDC) by phosphorylating pyruvate dehydrogenase E1α subunit (PDHA) [1]. There are four isoforms of PDHK (PHDK1-4) in human and PDC consists of three enzymes including PDHA, dihydrolipoamide acetyltransferase (E2 subunit), and lipoamide dehydrogenase (E3 subunit) [2,3]. The L2 domain of E2 is known to be the docking site of PDHKs [4]. PDC activity is crucial for maintaining oxidative phosphorylation (OXPHOS) by converting pyruvate to acetyl-CoA, the first step of the tricarboxylic acid (TCA) cycle [5]. Thus, inhibition of PDC activity is associated with many diseases, including lactic acidosis, diabetes, cerebrovascular and cardiovascular diseases, and cancer [4]. Cancer cells generally prefer glycolysis for producing energy rather than OXPHOS, even in O_2_ abundant conditions. This phenomenon is known as the Warburg effect [6]. Increased PDHK activity, which is often induced by oncogenic overexpression or post-translational modifications, has long been established as a molecular marker of aerobic glycolysis [7,8]. Hence, PDHK is being highlighted as a therapeutic target for developing anti-cancer drugs [9]. Several approaches, including structure-guided or computational studies, were attempted for developing novel inhibitors of PDHK from chemical compounds and natural products [10,11]. Diverse small molecules, such as dichloroacetate (DCA), AZD7545, JX06, radicinol, and phenylbutyrate (PB), are under ongoing investigation for their potential PDHK inhibitory activity [4,12]. However, there are only a few PDHK inhibitors, such as DCA and PB, available for clinical applications [4,13].

Huzhangoside A (Hu.A) is a triterpenoid glycoside isolated from *Anemone rivularis* Buch.-Ham. or *A. hupehensis* var. *japonica* (Thunb.) Bowles and Stearn (belonging to Ranunculaceae family). These plants were traditionally used for treating inflammation, pulmonary diseases, and malignant cancer [14,15,16]. Recently, we found that *A. rivularis* has a potent anti-tumor effect through inhibition of PDHK1 activity [17]. Although Hu.A was first isolated more than 30 years ago [14], its pharmacological activity is still not fully characterized. The cytotoxic effect of Hu.A on several human cancer cells, including HL-60, A549, HSC-2, and HSC-4, has been previously reported [16]. However, the molecular mechanism underlying cytotoxic action of Hu.A is still not elucidated. Therefore, we hypothesized that the anti-tumor properties of Hu.A might be due to the inhibition of PDHK activity. 

In this study, we demonstrated that Hu.A, isolated from *A. rivularis*, has an inhibitory action on PDHK1 activity and induces the apoptosis of cancer cells by decreasing the mitochondrial membrane potential. To the best of our knowledge, this is the first report providing experimental evidence of the anti-tumor effect of Hu.A and elucidating its mode of action.

## 2. Results

### 2.1. Hu.A Decreases Cancer Cell Viability and Inhibits PDHK Enzyme Activity

**Figure 1:**
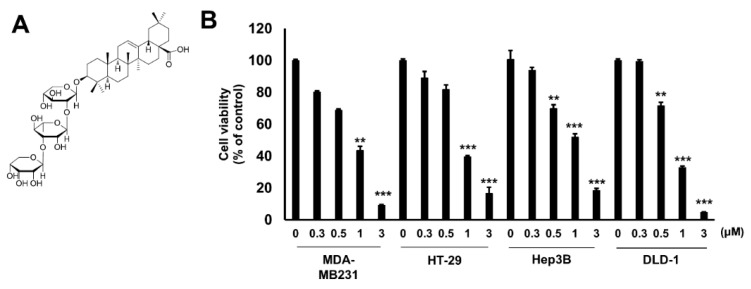
Huzhangoside A (Hu.A) decreased the cell viability of several cancer cell lines. (**A**) Chemical structure of Hu.A was indicated. (**B**) The indicated cells were treated with Hu.A for 24 h and 3-(4,5-dimethylthiazol-2-yl)-2,5-diphenyltetrazolium bromide (MTT) assay was performed. The cell viability is shown as mean ± standard deviations (SD). **, *p* < 0.01 and ***, *p* < 0.001 compared with control.

**Figure 2:**
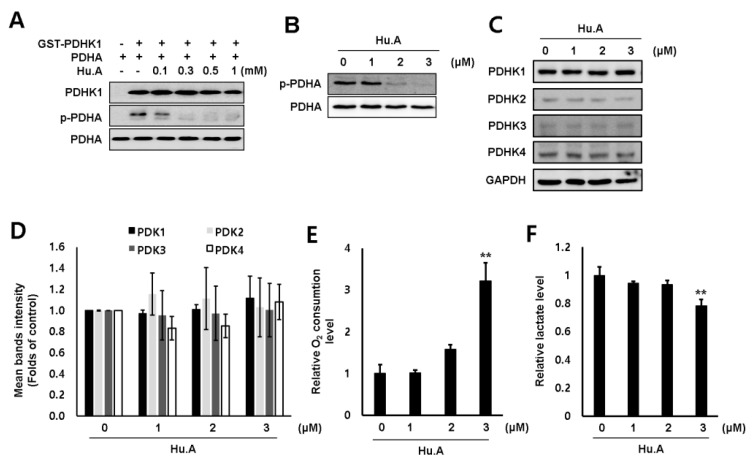
Hu.A reduced the PDHK1 activity and promoted oxidative phosphorylation (OXPHOS) in DLD-1 cells. (**A**) In vitro PDHK1 kinase assay was performed. (**B, C**) DLD-1 cells were treated with Hu.A in serum-free medium for 4 h. The levels of phosphorylated pyruvate dehydrogenase E1α subunit (PDHA) (**B**), and PDHK1-4 (**C**) were analyzed using western blot assay. PDHA (**B**) and GAPDH (**C**) were used as loading controls. (**D**) The intensity of bands (PDHK1-4/GAPDH) from three independent experiments was measured and indicated by mean ± SD. (**E**) DLD-1 cells were treated with Hu.A in serum-free medium for 6 h. O_2_ consumption rate was measured by using commercially available Oxygen Consumption Rate Assay Kit. (**F**) Lactate production was measured by lactate fluorometric assay kit (right panel). The data are shown as mean ± SD, respectively. **, *p* < 0.01 compared with control.

The cytotoxic effect of Hu.A in several human cancer cell lines, including MDA-MB-231, HT-29, Hep3B, and DLD-1 cells, was examined by 3-(4,5-dimethylthiazol-2-yl)-2,5-diphenyltetrazolium bromide (MTT) assay. The viability of these cells was significantly reduced upon Hu.A treatment (Figure 1B). The growth of LLC cells also decreased upon treatment with Hu.A (Supplementary Figure S1A). To investigate whether Hu.A has an inhibitory effect on the kinase activity of PDHK1, we performed an in vitro kinase assay using glutathione S-transferase (GST)-conjugated-PDHK1 and recombinant PDHA protein. In the Hu.A containing samples, the kinase activity of GST-PDHK1 was significantly inhibited (Figure 2A). The intracellular PDHK1 activity was measured using phospho-PDHA levels as a marker. We found that Hu.A treatment also inhibited the PDHK1 activity in DLD-1 and LLC cells (Figure 2B, and Supplementary Figure S1B). We also confirmed the protein expression of all these isozymes. The protein levels of these isozymes were very low and did not significantly change upon Hu.A treatment (Figure 2C,D). Since the inhibition of PDHK activity promotes the OXPHOS [18], we measured the O_2_ consumption rate and lactate production level upon Hu.A treatment. The O_2_ consumption rate was found to be increased and the secretory lactate levels in media were reduced upon Hu.A treatment in DLD-1 cells, as expected (Figure 2E,F).

### 2.2. Hu.A Inhibits PDHK Enzyme Activity by Binding to the ATP-Binding Pocket of PDHK1

**Figure 3:**
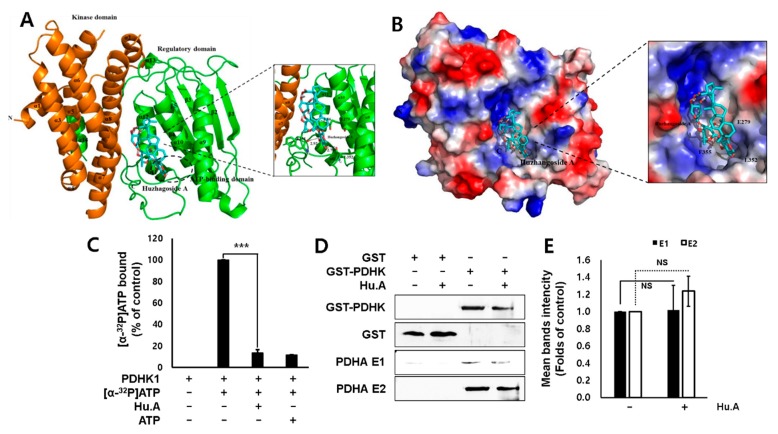
Hu.A interacted with the ATP-binding pocket of PDHK1. (**A**) The modeled structure of PDHK1 (PDB ID: 2Q8F) with Hu.A (CID: 73347426) around the ATP-binding domain is shown as a ribbon representation. The interaction residues between PDHK1 and Hu.A are shown, and hydrogen bonds are shown as black dotted lines. (**B**) The complex structure of PDHK1 with Hu.A was predicted as a surface representation. The relative distribution of the electrostatic surface of PDHK1 is shown with the acidic region in red, basic region in blue, and neutral region in white. (**C**) ATP-binding assay of PDHK1 in the presence or absence of Hu.A is shown. [α-^32^P]ATP-bound values were measured using scintillation counter. The results were calculated as percentage values in comparison to control and shown as mean ± SD. ***, *p* < 0.01 compared with positive control group (2nd lane). (**D**) PDC subunits and PDHK-binding assay was performed. (**E**) The bands intensity of panel (**D**) was indicated. The data are indicated as mean ± SD. NS, not significant.

To elucidate the mechanism of inhibition of PDHK activity by Hu.A, we constructed a structural model of PDHK1 and Hu.A interaction. The interaction and binding affinity of PDHK1 and Hu.A were analyzed in silico. The modeled structure of PDHK1 with Hu.A is shown as ribbon and surface representations (Figure 3A,B). The modeling results suggested that Hu.A can bind to the charged residue Glu^279^ and the hydrophobic residues Leu^352^ and Phe^355^ of PDHK1 in the complex. Hu.A was predicted to bind near the ATP-binding domain in the C-terminus of PDHK1, which is located around the two helices (α10 and α11) and the loop between α11 and α12. The data from in silico modeling of Hu.A binding to other PDHKs, including PDHK2-4, also demonstrated similar results with PDHK1 (Supplementary Figure S2). Therefore, an ATP-binding assay was used to confirm the binding ability of Hu.A to PDHK1. We found that the [α-^32^P]ATP-binding activity of PDHK1 was decreased in the presence of Hu.A (Figure 3C). However, Hu.A did not significantly affect the binding of PDHK and PDC E2 subunit (Figure 3D,E).

### 2.3. Hu.A Induces Mitochondrial ROS and Mitochondrial Damage in DLD-1 Cells

**Figure 4:**
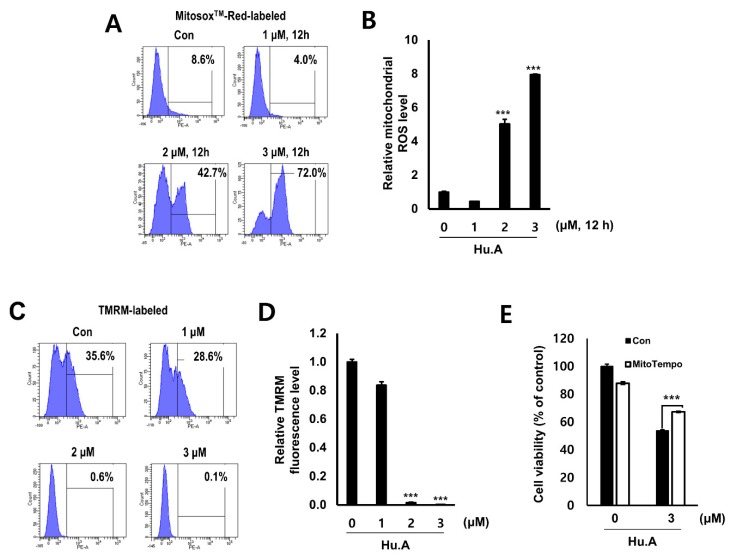
Hu.A induced mitochondrial reactive oxygen species (ROS) production and mitochondrial damage in DLD-1 cells. DLD-1 cells were treated with Hu.A for indicated time points in serum-free condition. The cells were stained with MitoSOX™ (Thermo Fisher Scientific, Rockford, IL, USA) Red (**A** and **B**), and tetramethylrhodamine methyl ester (TMRM) (**C** and **D**). The fluorescence for both assays was measured by FACS analysis. The relative mitochondrial ROS and TMRM fluorescence levels were calculated to determine the fold difference in comparison to control and are shown as mean ± SD. ***, *p* < 0.001. (**E**) In the serum-free condition, the cells were pre-treated with MitoTEMPO (Sigma-Aldrich, St. Louis, MO, USA) (50 μM) for 1 h and treated with 3 μM of Hu.A for 6 h. MTT assay was then performed. Data are indicated as mean ± SD. ***, *p* < 0.001 compared with the only Hu.A-treated group and MitoTEMPO with Hu.A co-treated group.

**Figure 5:**
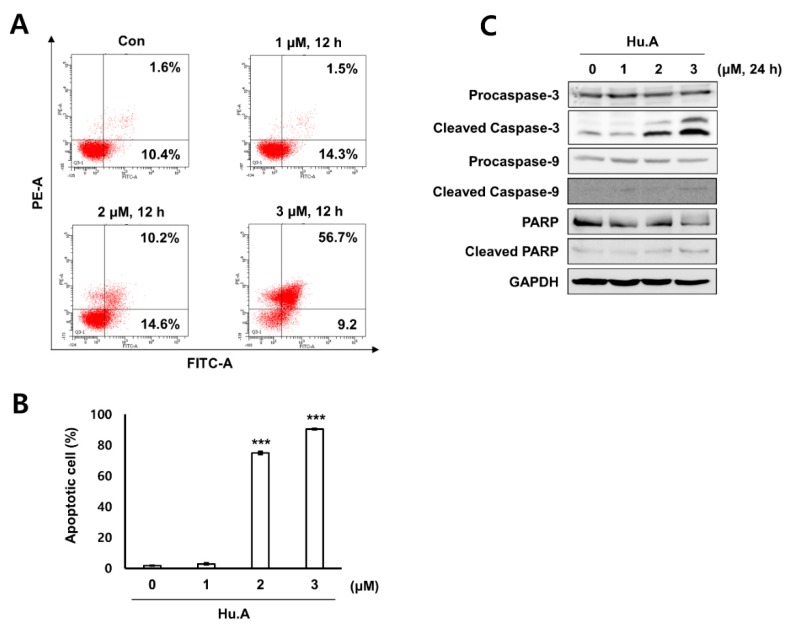
Hu.A induced apoptosis in DLD-1 cells. (**A**, and **B**) The cells were stained with annexin V-PI and were analyzed using FACS. The data are shown as mean ± SD, respectively. ***, *p* < 0.01 compared with control. (**C**) The expression of caspase-3, caspase-9, and poly ADP-ribose polymerase (PARP) in the cells was analyzed by western blot assay. GAPDH was used as a loading control.

Levels of mitochondrial ROS were measured by MitoSOX assay in Hu.A-treated DLD-1 cells. Mitochondrial ROS was markedly increased by Hu.A treatment at concentrations of 2 and 3 μM (Figure 4A,B). The mitochondrial depolarization was examined by tetramethylrhodamine methyl ester (TMRM) staining and the results showed that the mitochondrial membrane of the cells was significantly depolarized upon Hu.A treatment (Figure 4C,D). To confirm whether the cytotoxicity of Hu.A was related to the mitochondrial ROS, the viability of DLD-1 cells was examined by MTT assay upon treatment with MitoTEMPO, which is a specific scavenger of mitochondrial superoxide. In DLD-1 cells pre-treated with MitoTEMPO, the cytotoxic effect of Hu.A was significantly reduced (Figure 4E). In addition, results of the Annexin V and PI staining and the western blotting assay showed that Hu.A treatment significantly induces apoptosis in DLD-1 cells (Figure 5).

### 2.4. Hu.A Decreases Tumor Growth and Phospho-PDHA Levels In Vivo

**Figure 6:**
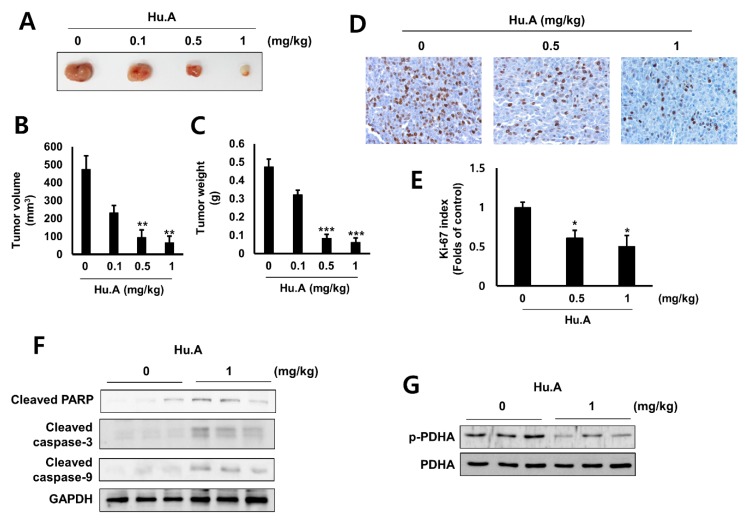
Hu.A reduced the tumor growth in vivo in LLC cell allograft mice model. The LLC cells were injected into the dorsal subcutaneous skin of C57BL/6 mice. On the 14th day, Hu.A at concentrations of 0.1, 0.5 and 1 mg/kg/day were intraperitoneally injected into the mice. (**A**) The representative picture of the tumors is shown. The tumor volume (**B**) and weight (**C**) were measured. Tumor volume and weight are indicated as mean ± SD. **, *p* < 0.01 and ***, *p* < 0.001 compared with the control group. (**D**) The tumor tissues were stained with the Ki-67 antibody and examined at ×200 magnification. (**E**) Ki-67 expression index was determined with Aperio Image scope software for three separate tumors. The data are shown as mean ± SD, respectively. *, *p* < 0.05 compared with control. (**F**) The expression of cleaved-caspase-3, caspase-9, and PARP in the tumor tissues were analyzed by western blot assay. GAPDH was used as a loading control. (**G**) The levels of phosphor-PDHA and total PDHA in the representative tumors were analyzed by western blot assay.

To investigate the anti-tumor effect of Hu.A in vivo, LLC murine lung cancer cells, treated with Hu.A, were injected into allograft mice models. Alanine aminotransferase (ALT), aspartate aminotransferase (AST) and blood urea nitrogen (BUN) levels in mice serum were not changed by Hu.A (Table 1). The creatinine concentrations were below the limit of detection in all groups. The growth of the allograft LLC cells was significantly suppressed by Hu.A administration (Figure 6A), in a dose-dependent manner, as observed by the decrease in the tumor volume (Figure 6B) and weight (Figure 6C). In addition, to evaluate the in vivo anti-tumor efficacy of Hu.A, the levels of Ki-67, a marker of cell proliferation, and activated forms of caspase-3, -9, and PARP, apoptosis markers, were examined in the tumor tissues. Immunohistochemistry results showed that staining of Ki-67 was reduced by Hu.A in the tumor tisss (Figure 6D). In the tumor tissues obtained from Hu.A-treated mice (1 mg/kg), the levels of cleaved forms of caspase-3, -9, and PARP were also increased by Hu.A (Figure 6F). Furthermore, the phosphor-PDHA levels were markedly decreased in the tumor tissues treated with Hu.A (Figure 6G). These results suggested that Hu.A suppressed the growth of the tumor and induced apoptosis in in vivo mice models through inhibiting PDHK1 activity.

## 3. Discussion

According to Warburg effect, many cancer cells prefer glycolysis followed by lactate fermentation rather than OXPHOS for energy production even under normoxic conditions [19]. PDHK is a key enzyme that regulates cellular bioenergetics and promotes aerobic glycolysis by inhibiting PDC activity [18]. Targeting the metabolic differences between cancer and normal cells has been recognized as a promising anti-cancer strategy [20]. Although heterogeneity exists in the metabolic ecology of tumor, reprogramming of pyruvate oxidation to lactate conversion is important in most of the hypoxic and aggressive cancers [21,22]. To shift OXPHOS to glycolysis, the inhibition of PDC by phosphorylating its E1α subunit mediated by PDHK is essential [1]. Among the four different isoforms of PDHK, PDHK1 is highly expressed in most cancers, especially in hypoxic conditions [7,23]. However, the expression of PDHK2-4 are variable depending upon the tumor types [8]. In addition, only PDHK1 is able to phosphorylate all three serine sites of PDH E1α subunit [24]. In this study, the levels of PDHK1 expression were much higher than that of other isoforms (Figure 2C). Hence, we focused on developing novel inhibitors of PDHK1 from natural product library. In this study, we report Hu.A as a novel PDHK1 inhibitor.

A previous study reported that the IC_50_ values of Hu.A against human cancer cell lines, such as HL-60, A549, HSC-2, and HSC-4, were 2.3, 1.5, 5.7, and 11.7 μM, respectively [16]. Similarly, our results showed that Hu.A has a cytotoxic effect of more than 80%, at a concentration of 3 μM, on MDA-MB-231, HT-29, Hep3B, DLD-1, and LLC cells (Figure 1B and Supplementary Figure S1A). Crystal structure analyses have demonstrated that the three major domains of PDHK, such as the N-terminal pyruvate-binding domain, lipoamide-binding domain, and C-term nucleotide-binding domain, play a key role in the regulation of PDHK activity [25,26]. In this study, Hu.A inhibited the binding of ATP with PDHK1 by interfering with its nucleotide-binding domain (Figure 3C). It was reported that aromatic DCA derivatives—JX06 and radicicol—interact with the ATP-binding pocket of PDHK1 [12,27]. The IC_50_ of JX06 and radicicol for inhibiting PDHK1 activity were determined as 49 nM and 400 μM, respectively. However, to suppress the intracellular PDHK1 activity, JX06 and radicicol were administered at much higher concentrations, that is, 10 μM [12,28]. In this study, we could not determine the precise IC_50_ of Hu.A for inhibiting PDHK1 activity, owing to the limitations of our kinase assay. However, the inhibition of intracellular PDHK1 activity was achieved at much lower concentrations of Hu.A, ranging from 2 to 3 μM (Figure 2B, and Supplementary Figure S1B). 

In cancer cells, inhibition of PDHKs increases the levels of electron-donor NADH in the mitochondria and the production of electron transport chain complex 1-based mitochondrial ROS [29]. As electron transport chain complex 1 has many ROS-sensitive components, these events can open the mitochondrial membrane potential-sensitive transition pore causing the release of proapoptotic factors leading to initiation of apoptosis [29]. In this study, Figure 4 and Figure 5 suggest that Hu.A induces apoptosis in cancer cells by inducing mitochondrial ROS. These results were also confirmed in an animal experiment (Figure 6). In addition, the concentration of Hu.A required for inhibition of tumor growth in in vivo experiments was also much lower than that of JX06, 40–80 mg/kg. Thus, Hu.A could be a more effective inhibitor of cancer than the previously reported synthetic PDHK inhibitors. The linearity range of dose-responsive effect of Hu.A to cancer cells is quite narrow, similar to several chemotherapeutic agents including mitoxantrone and paclitaxel [30]. In this study, we examined the general toxicity of Hu.A on the liver and kidney function by means of serum biochemical analysis (Table 1). However, to find out the safe therapeutic range of Hu.A, further extensive preclinical and clinical studies should be performed.

## 4. Materials and Methods

### 4.1. Materials

Antibodies against glyceraldehyde 3-phosphate dehydrogenase (GAPDH, #sc-32233), PDC E2 subunit (#sc-271352), PDHA (#sc-377092), and Ki67 (#sc-23900) were purchased from Santa Cruz Biotechnology (Dallas, TX, USA). Antibodies for caspase-3 (#9665s), -9 (#9508s) and PARP (#9542s) were supplied from Cell Signaling Technology (Danvers, MA, USA). Antibodies for PDHK1 (#ADI-KAP-PK112) and PDHK3 (#32581) were purchased from Enzo Life Sciences (Farmingdale, NY, USA) and NovusBio (Littleton, CO, USA), respectively. Antibodies against PDHK2 (#41330) and 4 (#38562) were purchased from Signalway Antibody (College Park, MD, USA). Antibody for phosphor-PDHA (#ab177461) was purchased from Abcam (Cambridge, UK). GST-PDHK1 construct was kindly provided from Jing Chen, Emory University and recombinant PDHA E1p clone was obtained from David Chuang, University of Texas.

### 4.2. Extraction and Isolation

*A. rivularis* plants were purchased at a folk medicine market, Yak-ryong-si, Daegu, Korea, in 2011. The plant material was identified by its morphological characteristics by Dr. J.W.S., a herbal specialist, and the voucher specimen (No. C308) was deposited at the National Development Institute of Korean Medicine (NIKOM). The dried plants (1.9 kg) were finely cut and extracted with methanol (MeOH) at room temperature for seven days, and the supernatant was evaporated under reduced pressure to give MeOH extracts (150.0 g). The crude MeOH extract was suspended in H_2_O (4.0 L) and then partitioned with different solvents, such as *n*-hexane (15.3 g), CH_2_Cl_2_ (14.8 g), EtOAc (43.2 g), and *n*-BuOH (72.1 g). The EtOAc-soluble extract (40.1 g) was loaded on a silica gel open column with a gradient system of EtOAc/MeOH/H_2_O (50:5:1 → 20:20:5, v/v/v) to yield 9 fractions (Fr. E1–E9). The fraction E4 was then subjected to reversed-phase medium pressure liquid chromatography (MPLC) using a gradient system of MeOH/H_2_O (70:30 → 100:0, v/v) to yield the active compound. The structure of the active compound was identified as Hu.A (Figure 1A) on the basis of various spectroscopic data and chemical evidence, which was in good agreement with those reported previously in the literature [14].

### 4.3. Cell Culture

DLD-1 and HT-29, human embryonic kidney HEK293T cells, and LLC cells were purchased from the American Type Culture Collection (Manassas, VA, USA). MDA-MB-231 cells and Hep3B cells were provided by Korean Cell Line Bank (Seoul, Korea). DLD-1, HEK293T, LLC and Hep3B cells were cultured in Dulbecco’s Modified Eagle’s Medium (DMEM; Welgene, Gyeongsan, Korea). HT-29 and MDA-MB-231 were maintained in RPMI 1640 medium (HyClone™, GE Healthcare Life Sciences, Logan, UT, USA). All culture media were supplemented with 10% (v/v) heat-inactivated fetal bovine serum (FBS, Welgene, Gyeongsan, Korea), and antibiotics (100 U/mL penicillin and 100 μg/mL streptomycin; Thermo Fisher Scientific, Rockford, IL, USA). The cells were maintained in a humidified 5% CO_2_ incubator at 37 °C.

### 4.4. Cell Viability Assay

The cytotoxic effect of Hu.A was confirmed using a MTT assay. Briefly, DLD-1 cells (10^5^ cells per well) were seeded in a 24-well plate and incubated overnight. The cells were then shifted to serum-free media and treated with Hu.A. MTT solution (2 mg/mL) was then added to the cells. The absorbance of formazan crystals formed were measured using a Spectramax M2 microplate reader (Molecular Devices, Sunnyvale, CA, USA) at 540 nm.

### 4.5. In Vitro PDHK Kinase Assay

The kinase assay of PDHK1 was carried out as described previously [17]. Briefly, HEK293T cells, transfected with GST-PDHK1 construct, were lysed, and the lysate was incubated with Glutathione Sepharose 4B beads (Amersham Biosciences, Uppsala, Sweden). The beads were washed and recombinant PDHA was added and incubated for 5 min at 30 °C in the kinase buffer. The phosphorylation of PDHA was analyzed by western blot analysis.

### 4.6. Western Blotting Assay

Western blotting assay was carried out as described previously [17]. Briefly, equal amounts (20 μg) of the cell lysates were separated by sodium dodecyl sulfate–polyacrylamide gel electrophoresis and were transferred onto the nitrocellulose membrane (Hybond ECL; GE Healthcare). The membrane was incubated with specific antibodies. The bands of the target proteins were detected using ECL Plus reagent and ImageQuant LAS 4000 (GE Healthcare, Menlo Park, CA, USA).

### 4.7. O_2_ Consumption Assay

The O_2_ consumption rate was determined using the Oxygen Consumption Rate Assay Kit (Cayman Chemical, Ann Arbor, MI, USA) following the manufacturer’s protocol. The fluorescence of the oxygen probe was measured using the Spectramax M2 microplate reader at wavelengths of 380/650 nm.

### 4.8. Lactate Production Assay

Lactate in the cell culture medium was analyzed by a lactate fluorometric assay kit (Biovision, Milpitas, CA, USA) [31] and measured using the Spectramax M2 microplate reader at 570 nm.

### 4.9. Structural Prediction of PDHK1 and Hu.A interaction

The crystal structure of PDHK1 protein was downloaded from the Protein Data Bank (ID: 2Q8F). The structure of Hu.A (CID: 73347426) compound was obtained from the NCBI PubChem Compound database. The structure of Hu.A was converted to an energy-minimized structure using OpenBabel in Pyrx. Research Design was used to computationally determine the potential activity and the binding affinity of Hu.A to PDHK1 protein. The structural prediction of PDHK1 and Hu.A complex was performed using AutoDock Vina in Pyrx. Among the complex models, the structure having the smallest binding energy was chosen. Binding affinity of the Hu.A to PDHK1 was −8.5 kcal/mol. The docking result was visualized using PyMOL.

### 4.10. ATP-Binding Assays

The cloned PDHK1 was expressed in *Escherichia coli* BL21(DE3). The overexpressed protein was purified using affinity chromatography and gel filtration chromatography. The purified His-tagged PDHK1 was bound to Ni-NTA beads in binding buffer (20 mM HEPES-K^+^ (pH 7.2) and 0.05% BSA) at 4 °C. The Ni-NTA beads with bound His-tagged PDHK1 were washed with the binding buffer followed by incubation with [α-^32^P]ATP, ATP, and Hu.A at 37 °C. The beads were then washed with wash buffer (PBS). The bead-bound PDHK1 protein was achieved by 250 mM imidazole, and its radioactivity was detected by liquid scintillation counting.

### 4.11. PDC Subunits and PDHK Binding Assay

HEK293T cells, transfected with GST-PDHK1 construct, were lysed and incubated with Glutathione Sepharose 4B beads. The beads were washed and HEK293T whole cell lysate was added with/without 500 μM of Hu.A and the samples were analyzed by western blot analysis.

### 4.12. Measurement of Mitochondrial Reactive Oxygen Species (ROS) and Mitochondrial Depolarization Assay

In the serum-free condition, DLD-1 cells (10^6^ cells per well) were treated with the indicated concentrations of Hu.A for 12 h. For mitochondrial ROS measurement, the cells were incubated with 5 μM MitoSOX™ Red for 1 h. For mitochondrial depolarization assay, the cells were treated with 50 nM tetramethylrhodamine methyl ester (TMRM; Thermo Fisher Scientific) for 30 min. Fluorescence intensities of these samples were measured using a BD FACSCANTO II (BD Biosciences, Sunnyvale, CA, USA) at the excitation and emission wavelengths of 510 and 580 nm, respectively.

### 4.13. Annexin V and Propidium Iodide (PI) Staining

DLD-1 cells (10^6^ cells per well) were incubated with the indicated concentration of Hu.A for 24 h in the serum-free media. The cells were stained using the Annexin V-FITC Apoptosis Detection kit (Life Technologies, Carlsbad, CA, USA), following the manufacturer’s protocol. Fluorescence intensities were analyzed using the BD FACSCANTO II.

### 4.14. Animals

C57BL/6 mice (male, six weeks old, weight 20–24 g) were purchased from Orient Bio Inc. (Sungnam, Korea) and housed in standard laboratory cages. The mice were maintained in a room at a constant temperature (22 ± 1 °C) and humidity (50% ± 5%) under a 12 h light/dark cycle. The mice were fed with a standard diet and drinking water ad libitum. All experimental procedures were performed in accordance with the Guidelines for the Care and Use of Laboratory Animals of the National Institutes of Health of Korea and were approved by the Institutional Animal Care and Use Committee of Pusan National University, Pusan, Korea (PNU-2018-1954).

### 4.15. Tumor Allograft and Drug Treatment

LLC cells (5 × 10^5^ cells/100 μL PBS) were injected into the dorsal subcutaneous skin of C57BL/6 mice (*n* = 8 mice per group). The control (untreated) group and Hu.A-treated groups were administered 0 mg/kg and 0.1 mg/kg, 0.5 mg/kg, and 1 mg/kg of Hu.A in 100 μL PBS/20 g via intraperitoneal (i.p.) injection for 14 days, respectively. The mice were killed 14 days after inoculation of LLC cells and the tumors were seceded from the mice and analyzed. The volumes of LLC cell derived-tumors were calculated using the formula ((length × width^2^)/2).

### 4.16. Immunohistochemistry

For immunohistochemical analysis, the tumor tissues were fixed with 3.7% formalin in PBS and embedded in paraffin. The paraffin sections were incubated with a primary antibody against the Ki-67, a cell proliferation marker, and visualized via Dako EnVision kit (Dako, Jena, Germany). The sections were counterstained with hematoxylin.

### 4.17. Blood Biochemistry

The blood sera were collected from the retro-orbital plexus of tumor-bearing mice treated with Hu.A. The levels of AST, ALT, BUN, and creatinine were analyzed by a commercial service provided by Green Cross Co. (Yongin, Korea).

### 4.18. Statistical Analysis

The values from the cell viability studies, and [α-^32^P] ATP-bound levels were calculated by the percentage of control groups and expressed as mean ± standard deviation (SD). The results from the studies on O_2_ consumption rate, mitochondrial ROS levels, TMRM fluorescence, volume and size of tumor tissues, and relative phosphor-PDHA levels in tumors were calculated as fold of control. The difference of mean values was analyzed by one-way analysis of variance with Tukey’s post-hoc test for comparing multiple groups and Student *t*-test for comparing two groups. All the experiments except for the animal studies were independently performed at least thrice.

## 5. Conclusions

In conclusion, Hu.A, a novel inhibitor of PDHK1 activity, reduced the growth of cancer cells by inducing mitochondrial ROS-mediated apoptosis. The in vivo efficacy of Hu.A was also confirmed in a murine allograft tumor model (Supplementary Figure S1C). Therefore, our studies suggest that Hu.A is a potent PDHK1 inhibitor and could be a novel candidate for developing anti-cancer drugs.

## Figures and Tables

**Table 1:** Effects in serum AST, ALT, BUN, and creatinine levels in Hu.A treated mice.

Concentration of Hu.A	ALT (U/L)	AST (U/L)	BUN (mg/dL)	Creatinine (mg/dL)
**Control**	14.8 ± 2.5	79.8 ± 35.3	8.6 ± 0.8	< 0.17
0.1 (mg/kg)	16.7 ± 2.5	94.5 ± 41.0	8.8 ± 0.9	< 0.17
0.5 (mg/kg)	19.2 ± 5.6	87.5 ± 33.8	8.9 ± 0.6	< 0.17
1 (mg/kg)	19.6 ± 3.1	95.2 ± 43.1	8.4 ± 1.0	< 0.17

Values are expressed as mean ± SD of six mice. ALT, alanine aminotransferase; AST, aspartate aminotransferase; BUN, blood urea nitrogen.

## Supplementary Materials

The following are available online at https://www.mdpi.com/2072-6694/11/5/712/s1, Figure S1: Hu.A inhibited cell viability and phosphorylation of PDHA in LLC cells, Figure S2: The interaction structures of PDHK isozymes with Hu. A are modeled.

## References

[1] Rardin M.J., Wiley S.E., Naviaux R.K., Murphy A.N., Dixon J.E. (2009). Monitoring phosphorylation of the pyruvate dehydrogenase complex. Anal. Biochem..

[2] Popov K.M., Kedishvili N.Y., Zhao Y., Shimomura Y., Crabb D.W., Harris R.A. (1993). Primary structure of pyruvate dehydrogenase kinase establishes a new family of eukaryotic protein kinases. J. Biol. Chem..

[3] Gudi R., Melissa M.B.-K., Kedishvili N.Y., Zhao Y., Popov K.M. (1995). Diversity of the pyruvate dehydrogenase kinase gene family in humans. J. Biol. Chem..

[4] Stacpoole P.W. (2017). Therapeutic targeting of the pyruvate dehydrogenase complex/pyruvate dehydrogenase kinase (PDC/PDK) axis in cancer. J. Natl. Cancer Inst..

[5] Zhang S., Hulver M.W., McMillan R.P., Cline M.A., Gilbert E.R. (2014). The pivotal role of pyruvate dehydrogenase kinases in metabolic flexibility. Nutr. Metab..

[6] López-Lázaro M. (2008). The warburg effect: Why and how do cancer cells activate glycolysis in the presence of oxygen?. Anti-Cancer Agents Med. Chem..

[7] Kim J.-W., Tchernyshyov I., Semenza G.L., Dang C.V. (2006). HIF-1-mediated expression of pyruvate dehydrogenase kinase: A metabolic switch required for cellular adaptation to hypoxia. Cell Metab..

[8] Saunier E., Benelli C., Bortoli S. (2016). The pyruvate dehydrogenase complex in cancer: An old metabolic gatekeeper regulated by new pathways and pharmacological agents. Int. J. Cancer.

[9] Sutendra G., Michelakis E.D. (2013). Pyruvate dehydrogenase kinase as a novel therapeutic target in oncology. Front. Oncol..

[10] Tso S.-C., Qi X., Gui W.-J., Wu C.-Y., Chuang J.L., Wernstedt-Asterholm I., Morlock L.K., Owens K.R., Scherer P.E., Williams N.S. (2014). Structure-guided development of specific pyruvate dehydrogenase kinase inhibitors targeting the ATP-binding pocket. J. Biol. Chem..

[11] Zhou X., Yu S., Su J., Sun L. (2016). Computational study on new natural compound inhibitors of pyruvate dehydrogenase kinases. Int. J. Mol. Sci..

[12] Sun W., Xie Z., Liu Y., Zhao D., Wu Z., Zhang D., Lv H., Tang S., Jin N., Jiang H. (2015). JX06 selectively inhibits pyruvate dehydrogenase kinase PDK1 by a covalent cysteine modification. Cancer Res..

[13] Michelakis E.D., Sutendra G., Dromparis P., Webster L., Haromy A., Niven E., Maguire C., Gammer T.L., Mackey J.R., Fulton D. (2010). Metabolic modulation of glioblastoma with dichloroacetate. Sci. Transl. Med..

[14] Mizutani K., Ohtani K., Wei J.-X., Kasai R., Tanaka O. (1984). Saponins from Anemone rivularis. Planta Med..

[15] Zhao C.-C., Shao J.-H., Fan J.-D. (2012). A new triterpenoid with antimicrobial activity from Anemone rivularis. Chem. Nat. Compd..

[16] Yokosuka A., Sano T., Hashimoto K., Sakagami H., Mimaki Y. (2009). Triterpene glycosides from the whole plant of Anemone hupehensis var. japonica and their cytotoxic activity. Chem. Pharm. Bull..

[17] Chung T.-W., Lee J.H., Choi H.-J., Park M.-J., Kim E.-Y., Han J.H., Jang S.B., Lee S.-O., Lee S.W., Hang J. (2017). Anemone rivularis inhibits pyruvate dehydrogenase kinase activity and tumor growth. J. Ethnopharmacol..

[18] Hitosugi T., Fan J., Chung T.W., Lythgoe K., Wang X., Xie J., Ge Q., Gu T.L., Polakiewicz R.D., Roesel J.L. (2011). Tyrosine phosphorylation of mitochondrial pyruvate dehydrogenase kinase 1 is important for cancer metabolism. Mol. Cell.

[19] Vander Heiden M.G., Cantley L.C., Thompson C.B. (2009). Understanding the Warburg effect: The metabolic requirements of cell proliferation. Science.

[20] Martinez-Outschoorn U.E., Peiris-Pages M., Pestell R.G., Sotgia F., Lisanti M.P. (2017). Cancer metabolism: A therapeutic perspective. Nat. Rev. Clin. Oncol..

[21] Parks S.K., Chiche J., Pouyssegur J. (2013). Disrupting proton dynamics and energy metabolism for cancer therapy. Nat. Rev. Cancer.

[22] Vlassenko A.G., McConathy J., Couture L.E., Su Y., Massoumzadeh P., Leeds H.S., Chicoine M.R., Tran D.D., Huang J., Dahiya S. (2015). Aerobic Glycolysis as a Marker of Tumor Aggressiveness: Preliminary Data in High Grade Human Brain Tumors. Dis. Markers.

[23] Papandreou I., Cairns R.A., Fontana L., Lim A.L., Denko N.C. (2006). HIF-1 mediates adaptation to hypoxia by actively downregulating mitochondrial oxygen consumption. Cell Metab..

[24] Kolobova E., Tuganova A., Boulatnikov I., Popov K.M. (2001). Regulation of pyruvate dehydrogenase activity through phosphorylation at multiple sites. Biochem. J..

[25] Jeoung N.H. (2015). Pyruvate Dehydrogenase Kinases: Therapeutic Targets for Diabetes and Cancers. Diabetes Metab. J..

[26] Roche T.E., Hiromasa Y. (2007). Pyruvate dehydrogenase kinase regulatory mechanisms and inhibition in treating diabetes, heart ischemia, and cancer. Cell. Mol. Life Sci..

[27] Kato M., Li J., Chuang J.L., Chuang D.T. (2007). Distinct structural mechanisms for inhibition of pyruvate dehydrogenase kinase isoforms by AZD7545, dichloroacetate, and radicicol. Structure.

[28] He Y., Li Y., Zhang S., Perry B., Zhao T., Wang Y., Sun C. (2013). Radicicol, a heat shock protein 90 inhibitor, inhibits differentiation and adipogenesis in 3T3-L1 preadipocytes. Biochem. Biophys. Res. Commun..

[29] Bonnet S., Archer S.L., Allalunis-Turner J., Haromy A., Beaulieu C., Thompson R., Lee C.T., Lopaschuk G.D., Puttagunta L., Bonnet S. (2007). A mitochondria-K+ channel axis is suppressed in cancer and its normalization promotes apoptosis and inhibits cancer growth. Cancer Cell.

[30] Porrata L.F., Adjei A.A. (2001). The pharmacologic basis of high dose chemotherapy with haematopoietic stem cell support for solid tumours. Br. J. Cancer.

[31] Fan J., Hitosugi T., Chung T.-W., Xie J., Ge Q., Gu T.-L., Polakiewicz R.D., Chen G.Z., Boggon T.J., Lonial S. (2011). Tyrosine phosphorylation of lactate dehydrogenase a is important for NADH/NAD+ redox homeostasis in cancer cells. Mol. Cell. Biol..

