# Supplementary material for: Huzhangoside A Suppresses Tumor Growth through Inhibition of Pyruvate Dehydrogenase Kinase Activity
Source: Cancers (Basel). 2019 May 23;11(5):712. doi: 10.3390/cancers11050712 (PMC6562422; doi:10.3390/cancers11050712)
Supplement: Supplementary file 1 [file cancers-11-00712-s001.pdf]

Supplementary materials Huzhangoside A Suppresses Tumor Growth through Inhibition of Pyruvate Dehydrogenase Kinase Activity

Choong-Hwan Kwak, Jung-Hee Lee, Eun-Yeong Kim, Chang Woo Han, Keuk-Jun Kim, Hanna Lee, MyoungLae Cho, Se Bok Jang, Cheorl-Ho Kim, Tae-Wook Chung and Ki-Tae Ha

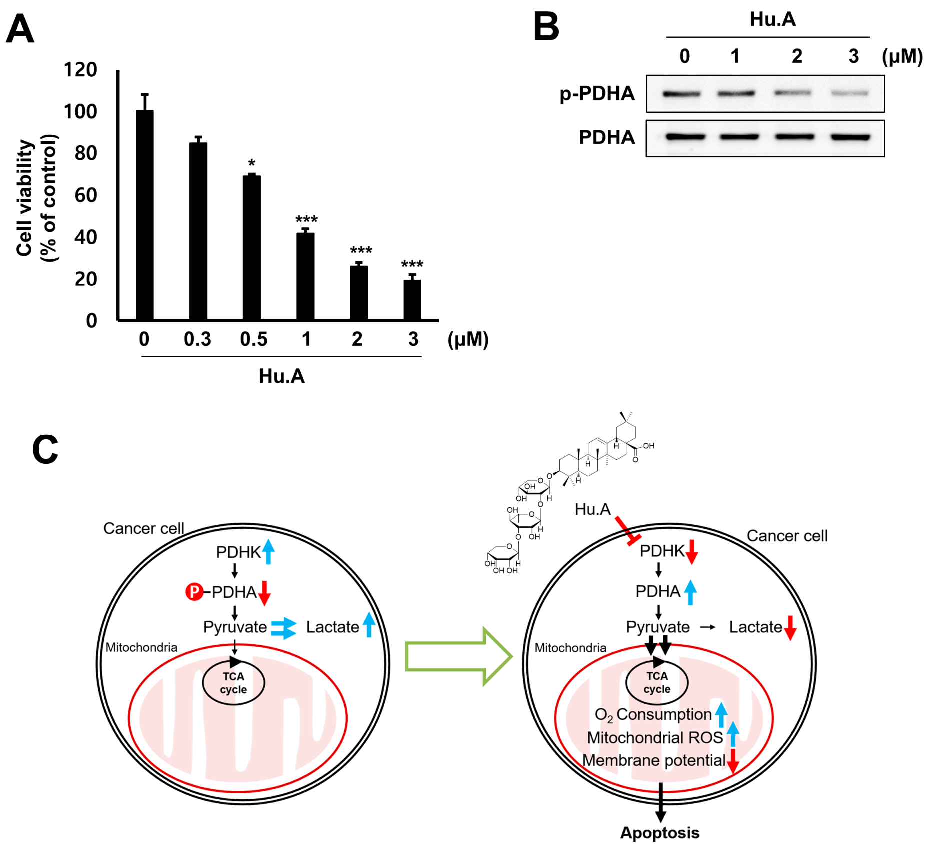

Figure S1. Hu.A inhibited cell viability and phosphorylation of PDHA in LLC cells. **(A)** The LLC cells were treated with indicated concentrations of Hu.A in serum free medium for 24 h and MTT assay was performed. The data was indicated as mean ± SD. * $p < 0.05$ and *** $p < 0.001$ compared with the control. **(B)** In serum free condition, the LLC cells were treated with indicated concentrations of Hu.A for 4 h. The levels of phosphor-PDHA and total PDHA were analyzed by Western blotting assay. **(C)** Schematic diagram of the mechanisms of Hu.A-mediated apoptosis was shown. The red circle and white P indicated phosphates.

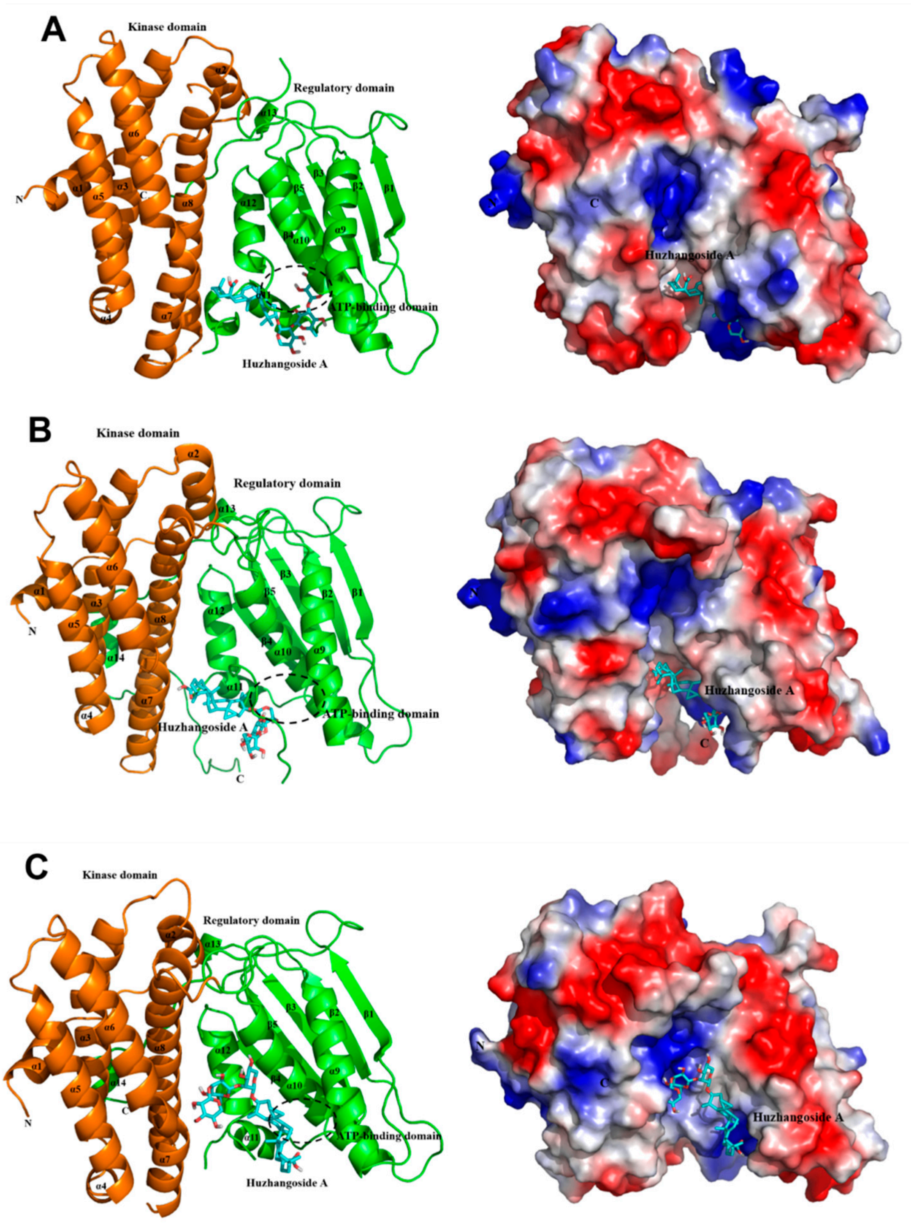

Figure S2. The interaction structures of PDHK isozymes with Hu. A are modeled. (A) PDHK2 (PDB ID: 5M4P) interacts with Hu. A (CID: 73347426) around the ATP-binding domain. The ribbon (left) and surface (right) representations are shown. **(B)** PDHK3 (PDB ID: 2PNR) and **(C)** PDHK4 (PDB ID: 2ZDX). The relative distribution of the electrostatic surface of PDHK isozyme is shown with the acidic region in red, basic region in blue and neutral region in white.

© 2019 by the authors. Licensee MDPI, Basel, Switzerland. This article is an open access article distributed under the terms and conditions of the Creative Commons Attribution (CC BY) license (http://creativecommons.org/licenses/by/4.0/).
